# HMG-CoA Reductase Inhibitors (Statins) May Preserve Hepatic Function and Reduce Portal-Systemic Shunting in Compensated Advanced Chronic Liver Disease: Results From the SHUNT-V Study

**DOI:** 10.14309/ctg.0000000000000980

**Published:** 2026-01-26

**Authors:** Robert S. Rahimi, Edward Mena, Kathryn J. Lucas, Michael P. McRae, John Kittelson, Joanne C. Imperial, Alastair D. Smith, Gregory T. Everson, for the SHUNT-V Subjects, Investigators, and Coordinators, Kiran Bambha

**Affiliations:** 1 Baylor Scott and White Health, Dallas, Texas, USA;; 2 California Liver Research Institute, Pasadena, California, USA;; 3 Lucas Research, Morehead City, North Carolina, USA;; 4 Custom DX Solutions LLC, Houston, Texas, USA;; 5 Consultant to HepQuant, LLC, Denver, Colorado, USA;; 6 HepQuant, LLC, Denver, Colorado, USA;; 7 Syneos Health, Morrisville, North Carolina, USA.

**Keywords:** cholate, liver function, metabolic dysfunction-associated steatohepatitis, chronic hepatitis C, portal hypertension

## Abstract

**INTRODUCTION:**

Factors associated with decline of hepatic function and increase in portal-systemic shunting, which herald clinical outcome in persons with compensated cirrhosis, are poorly characterized. We used cholate challenge to evaluate the associations of liver disease etiology, concomitant diabetes, and maintenance drug therapy, with the degree of hepatic dysfunction and portal-systemic shunting.

**METHODS:**

In the SHUNT-V study, there were 255 subjects with compensated (Child-Pugh class A) cirrhosis who underwent cholate challenge, involving oral administration of [2,2,4,4-^2^H] cholate and measurement of its serum concentrations at 20 and 60 minutes. Test outputs included a disease severity index (DSI) to assess global liver function and SHUNT% to assess portal systemic shunting.

**RESULTS:**

Eighty-seven percent of subjects were overweight, 65% were obese, 48% had metabolic dysfunction-associated steatotic liver disease (MASLD)/metabolic dysfunction-associated steatohepatitis (MASH), 51% had type 2 diabetes mellitus, 49% were taking anti-diabetic drugs, and 45% were taking lipid-lowering drugs. Laboratory values and clinical scores of MASLD/MASH subjects were similar to subjects with other etiologies for liver disease. In univariable regression, MASLD/MASH, diabetes mellitus, metformin, and statins were associated with lower DSI and SHUNT%. In multivariable regression, lower DSI was attributable to statins (*P* = 0.0354) and metformin (*P* = 0.0561). The combined use of lipid-lowering and anti-diabetic drugs, compared with no use, was associated with 19% reduction in DSI.

**CONCLUSION:**

Concomitant use of statins alone or in combination with metformin was independently associated with preserved hepatic function (DSI) and reduced portal-systemic shunting (SHUNT%).

## Graphical abstract

**Figure:**
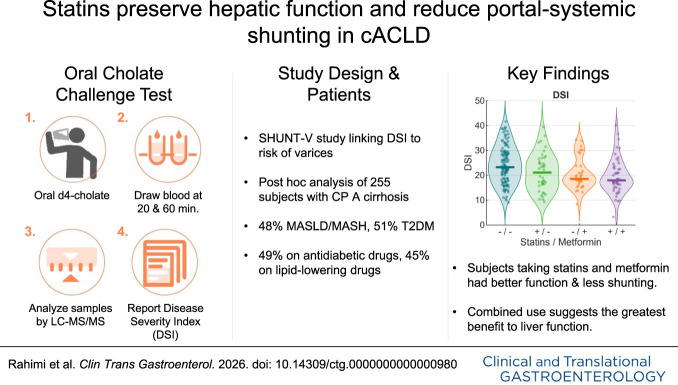

## INTRODUCTION

Over 100 million Americans are at risk for, and 15–30 million have, chronic liver disease (CLD) (1). CLD is the fourth leading cause of death in the age range from 45 to 64 years and the sixth leading cause of death in the age range from 25 to 44 years (2). Inpatient hospitalization costs related to CLD rose from $14.9 billion in 2012 to $18.8 billion in 2016 (3). The clinical and economic impact of CLD on the US healthcare system can only be dampened by early detection and effective intervention.

Given the enormous numbers of Americans at risk and who have CLD, effective and low-cost interventions are sorely needed. Statins may fulfill this unmet need because they seem to be safe in early and late stages of fibrosis, including compensated cirrhosis (4). Several studies have suggested potential beneficial effects of statins in CLD regardless of etiology (5). These studies have shown reduction in portal hypertension, lower rate of decompensating events, lower incidence of hepatocellular carcinoma, lower rate of infectious complications, and increased survival. However, long-term prospective trials are still needed to confirm these findings. Although the mechanisms whereby statins achieve these clinical benefits are unknown, 2 plausible explanations could include preservation of liver function and reduction in progression of fibrosis and portal hypertension.

SHUNT-V was a study of subjects with advanced CLD who underwent dual cholate shunt and oral cholate challenge testing and screening endoscopy (6,7). The subjects had a high prevalence of overweight body habitus, obesity, metabolic dysfunction-associated steatohepatitis (MASH), diabetes mellitus (DM), and use of both antidiabetic and lipid-lowering drug therapies. For these reasons, we used the SHUNT-V cohort to examine the associations of metabolic dysfunction-associated steatotic liver disease (MASLD)/MASH diagnosis, concomitant DM, and use of maintenance drug therapy with severity of hepatic dysfunction and portal-systemic shunting.

## METHODS

The study design and primary results of the SHUNT-V study have been reported previously (6,7). Twenty-seven US clinical centers participated and enrolled subjects from January 2019 through May 2021. The study was conducted according to the Declaration of Helsinki and guidelines for Good Clinical Practice defined by International Council on Harmonization. Research subjects were recruited from the centers' hepatology clinics and through posted notices within the centers' domains. All subjects gave written informed consent to participate in the study. The study was approved by the Institutional Review Boards (IRBs) of the respective institutions, registered at ClinicalTrials.gov, NCT03583996, and the cholate compounds were administered under a US FDA-issued Investigational Device Exemption, G180098/S002.

The study subjects were adults who had been selected for screening or surveillance endoscopy as part of their standard of care (see Supplementary Figure 1, Supplementary Digital Content, http://links.lww.com/CTG/B453). The 255 subjects with valid SHUNT tests had compensated Child-Pugh class A cirrhosis without ascites, encephalopathy, or history of variceal hemorrhage. The inclusion criteria included suspected or definite cirrhosis as determined by prior liver biopsy, radiologic (including elastography) or clinical criteria, or chronically abnormal liver tests with low platelet count. The major exclusions were Child-Pugh B and C cirrhosis, refractory ascites or encephalopathy, prior variceal hemorrhage, known large varices, or endoscopic or surgical treatment of varices. Recipients of a transplanted liver were also excluded. Each subject completed medical history, physical examination, and standard laboratory tests (aspartate transaminase, alanine transaminase, alkaline phosphatase, bilirubin, albumin, international normalized ratio, and complete blood count). Disease etiology was assigned by the investigators at each clinical center based on historical and laboratory data. The diagnosis of MASLD/MASH was established by clinical criteria with established risk factors for MASLD (obesity, diabetes, etc.) and ruling out other etiologies. Stage of liver disease was assessed by clinical, radiological, and laboratory criteria (MELD, MELD-Na, and Child-Pugh scores), and where available, by liver biopsy or elastography.

Additional exclusions to participation included ongoing alcohol consumption of more than 50 g/d, decompensated liver disease, current malignancy, history of angina, myocardial infarction, congestive heart failure, pregnancy or intent to become pregnant, and inability to provide informed consent. Patients with chronic kidney disease stage 4 or 5 (glomerular filtration rate < 30 mL/min/1.73 m^2^), extensive small bowel resection, or severe gastroparesis were excluded.

Study data were collected and monitored by the contract research organization for the study, Syneos Health (Morrisville, NC).

### Dual cholate test procedure and parameters

The details of the dual cholate shunt test (HepQuant SHUNT) and the oral cholate challenge test (HepQuant DuO) have been reported elsewhere and are summarized below (8–10). All patients were studied after fasting, either overnight or for more than 5 hours. A 20 mg dose of [24-^13^C]cholic acid (13C-CA) and albumin solution was injected intravenously over 1 minute; at the same time, a 40 mg dose of [2,2,4,4-^2^H]cholic acid (d4-CA) was administered orally. Blood samples for measurement of cholate concentrations were drawn through the indwelling catheter before dosing and 5 ± 1, 20 ± 2, 45 ± 5, 60 ± 5, and 90 ± 5 minutes after dosing. Serum was separated, placed in transport tubes, and shipped to the HepQuant, LLC lab, for analysis.

The dual cholate shunt test (V1.0) involves both intravenous and oral dosing of cholate isotopes and 6 timed peripheral venous blood samples (3 mL each) (11). The oral cholate challenge test involves only an oral dose of cholate isotopes and 2 timed peripheral venous blood samples collected at 20 and 60 minutes (10,12). The dose divided by area under the oral [d4-CA] concentration vs time curve defines the portal clearance, and the dose divided by area under the intravenous [13C-CA] concentration vs time curve defines the systemic clearance. In the oral cholate challenge test, the intravenous clearance is derived rather than measured. In this study, we report the results with the oral cholate challenge test version. Refer to the Supplementary Materials (see Supplementary Digital Content, http://links.lww.com/CTG/B453) for results with the dual cholate shunt test versions.

The clearance values from the cholate challenge tests are used to generate the test parameters summarized below.

- Systemic hepatic filtration rate (HFR) is the clearance (dose/AUC) of the intravenously administered 13C-cholate adjusted for body weight.
- Portal HFR is the clearance (dose/AUC) of the orally administered d4-cholate adjusted for body weight.
- Disease severity index (DSI) is a score of disease severity based on systemic and portal HFRs indexed to the maximum HFRs of healthy controls (0, no disease; 50, end-stage disease).
- Hepatic reserve is a percentage of liver health based on systemic and portal HFRs, similar to DSI, but instead indexed to the means (−1 SD) in healthy controls with lean body mass (100%, normal reserve; 0%, no reserve).
- SHUNT% is a percentage of cholate shunting from portal to systemic circulation, calculated from the ratio of systemic to portal HFRs.

### Statistical analysis

We compared patient demographics, laboratory values, and cholate challenge test parameters by subjects with MASLD/MASH vs other liver disease etiologies, and subjects with DM vs those without DM. Continuous variables were reported as mean ± SD, and differences between patient groups were analyzed for significance using the t test. Categorical data were reported as counts or percentages, and differences between patient groups were analyzed for significance using the χ^2^ test. In all analyses, statistical significance was set at *P* < 0.05. Given the exploratory nature of this analysis, *P*-values were not adjusted for multiple comparisons. The results should therefore be interpreted as hypothesis-generating rather than confirmatory. Univariate and multivariable linear regression analyses tested the association of DSI, SHUNT%, and other cholate challenge test variables with diagnosis of MASLD/MASH, diagnosis of DM, use of antidiabetic drug therapy, use of lipid-lowering therapy, and with specific drugs, such as metformin and HMG CoA reductase inhibitors (statins). Information on the indication, dose, or duration was not systematically collected. Associations between cholate challenge test parameters and treatment subgroups (i.e., antidiabetic drug therapy alone, lipid-lowering therapy alone, both in combination, or neither) were assessed by analysis of variance.

## RESULTS

### Study population

Selected characteristics of the overall study population are reported in Supplementary Table S1 (see Supplementary Digital Content, http://links.lww.com/CTG/B453). The distribution by race/ethnicity was 91% White, 6% Black or African American, 1% Asian, 2% other race, and 15% Hispanic ethnicity. The average age was 61 ± 11 years, weight 95 ± 23 kg, and body mass index (BMI) 33 ± 7 kg m^−2^. Fifty percent were male, 87% overweight, and 65% obese. Forty-eight percent had MASLD/MASH, 51% had DM, 49% were taking antidiabetic drugs, and 45% were taking lipid-lowering drugs, mainly statins. The statins used by these subjects were atorvastatin (n = 43), rosuvastatin (n = 26), simvastatin (n = 12), pravastatin (n = 13), lovastatin (n = 1), and pitavastatin (n = 1). Seventy-seven (32%) had small esophageal varices, and 28 (12%) had large esophageal varices at the protocol-specified screening or surveillance endoscopy.

### Comparison of the characteristics of MASLD/MASH vs non-MASLD/MASH subjects

The characteristics of MASLD/MASH, vs other etiologies of CLD, are given in Table 1. MASLD/MASH subjects were more likely to be female (*P* = 0.0006), had higher BMI (*P* = 0.0012), and a greater percentage were overweight (*P* = 0.0002), had obesity (*P* = 0.0001), or had diabetes (*P* < 0.0001). Fewer were Black or African American (*P* = 0.0127). The prevalences of both small and large esophageal varices were similar. MASLD/MASH subjects were more likely to be taking antidiabetic or lipid-lowering drugs, particularly statins, metformin, and sulfonylureas. Standard blood tests, Child-Pugh score, and MELD score were similar, and means for the blood tests were in the normal range (Table 2).

**Table 1.** Subject characteristics by MASLD/MASH diagnosis

Characteristics	Other chronic liver disease etiologies		MASLD/MASH		*P* value
	n	Mean ± SD or n (%)	n	Mean ± SD or n (%)	
Age, yr	133	60.1 ± 10.5	122	61.7 ± 10.8	0.2452
Male	133	80 (60.2%)	122	47 (38.5%)	**0.0006**
Weight, kg	133	93.5 ± 25.5	122	97.7 ± 20.4	0.1415
BMI, kg m^−2^	133	32.1 ± 7.8	122	35.0 ± 6.0	**0.0012**
Overweight	133	106 (79.7%)	122	117 (95.9%)	**0.0002**
Obese	133	71 (53.4%)	122	94 (77.1%)	**0.0001**
DM	133	43 (32.3%)	122	87 (71.3%)	**<0.0001**
Race					
White	133	116 (87.2%)	122	116 (95.1%)	**0.0487**
Black or African American	133	13 (9.8%)	122	2 (1.6%)	**0.0127**
Asian	133	1 (0.8%)	122	1 (0.8%)	1.0000
Other	133	3 (2.3%)	122	3 (2.5%)	1.0000
Ethnicity					
Hispanic	133	21 (15.8%)	122	17 (13.9%)	0.8107
Non-Hispanic	133	112 (84.2%)	122	105 (86.1%)	0.8107
Presence of esophageal varices					
Small varices	124	42 (31.6%)	118	35 (28.7%)	0.5722
Large varices	124	13 (9.8%)	118	15 (12.3%)	0.7334
Antidiabetic and lipid-lowering drugs					
Statins	133	32 (24.1%)	122	64 (52.5%)	**<0.0001**
Metformin	133	25 (18.8%)	122	62 (50.8%)	**<0.0001**
Sulfonylureas	133	11 (8.3%)	122	36 (29.5%)	**<0.0001**
GLP-1 analog	133	10 (7.5%)	122	22 (18.0%)	**0.0329**
Pioglitazone	133	3 (2.3%)	122	11 (9.0%)	**0.0364**
SGLT-2 inhibitor	133	10 (7.5%)	122	24 (19.7%)	**0.0139**
DPP-4 inhibitor	133	2 (1.5%)	122	9 (7.4%)	**0.0458**
Insulin	133	12 (9.0%)	122	37 (30.3%)	**<0.0001**
Vitamin E	133	4 (3.0%)	122	8 (6.6%)	0.2978
Nonselective beta-blockers	133	14 (10.53%)	122	14 (11.48%)	0.9668
Carvedilol	133	5 (3.76%)	122	4 (3.28%)	1.0000
Propranolol	133	7 (5.26%)	122	6 (4.92%)	1.0000
Nadolol	133	2 (1.50%)	122	4 (3.28%)	0.6027

BMI, body mass index; DM, antidiabetic drugs; MASH, metabolic dysfunction-associated steatohepatitis; MASLD, metabolic dysfunction-associated steatotic liver disease.

Bold values indicate statistical significance (*P* < 0.05).

**Table 2.** Laboratory tests, clinical scores, and cholate challenge test results by MASLD/MASH diagnosis

	Other chronic liver disease etiologies		MASLD/MASH		*P* value
	n	Mean ± SD or n (%)	n	Mean ± SD or n (%)	
Laboratory values					
Albumin, g dL^−1^	133	4.2 ± 0.4	120	4.3 ± 0.4	0.2853
Alk. Phos., U L^−1^	133	101.0 ± 58.4	119	100.4 ± 49.4	0.9369
ALT, U L^−1^	131	38.4 ± 39.9	118	37.8 ± 24.1	0.8922
AST, U L^−1^	127	41.7 ± 29.4	117	42.3 ± 19.9	0.8618
Bilirubin, mg dL^−1^	130	0.8 ± 0.5	119	0.7 ± 0.5	0.1691
Creatinine, mg dL^−1^	133	0.9 ± 0.3	120	0.9 ± 0.3	0.8616
INR	131	1.1 ± 0.1	114	1.1 ± 0.2	0.6871
Platelets, ×10^3^ μL^-1^	128	156 ± 70	118	152 ± 67	0.6355
Clinical scores					
Child-Pugh score	133	5.2 ± 0.4	122	5.1 ± 0.3	0.2475
MELD score	130	7.9 ± 2.1	113	7.8 ± 2.3	0.7087
Cholate challenge test					
DSI	133	23.0 ± 8.0	122	21.5 ± 6.9	0.1015
SHUNT%, %	133	40.6 ± 16.6	122	34.9 ± 13.5	**0.0029**
Hepatic reserve, %	133	69.9 ± 20.0	122	74.6 ± 17.9	0.0528
HFR_P_, mL min^−1^ kg^−1^	133	10.8 ± 7.3	122	11.9 ± 6.6	0.1778
HFR_S_, mL min^−1^ kg^−1^	133	3.4 ± 1.0	122	3.4 ± 0.8	0.9703

Bold values indicate statistical significance (*P* < 0.05).

Alk. Phos., alkaline phosphatase; ALT, alanine transaminase; AST, aspartate transaminase; DSI, disease severity index; HFR_P_, portal hepatic filtration rate; HFR_S_, systemic hepatic filtration rate; INR, international normalized ratio; MASH, metabolic dysfunction-associated steatohepatitis; MASLD, metabolic dysfunction-associated steatotic liver disease; MELD, model for end-stage liver disease.

Cholate challenge test results in MASLD/MASH vs non-MASLD/MASH subjects are given in Table 2. We had anticipated that MASLD/MASH subjects would have worse function and shunting due to the high prevalence of obesity and DM, but portal HFR (*P* = 0.18) and hepatic reserve (*P* = 0.05) trended higher, DSI (*P* = 0.10) trended lower, and SHUNT% (*P* < 0.003) was significantly lower in MASLD/MASH subjects. These results indicated, surprisingly, that the MASLD/MASH cohort had better liver function and less portal-systemic shunting compared with the non-MASLD/MASH cohort.

### Comparison of the characteristics of diabetic (DM) vs non-DM subjects

The characteristics of DM vs non-DM subjects are given in Table 3. DM subjects had similar gender distribution but older mean age (*P* = 0.0020) and greater mean BMI (*P* = 0.0043), percentage who were overweight (*P* = 0.0002), and percentage who were obese (*P* = 0.0280). MASLD/MASH was the dominant etiology in DM subjects, followed by hepatitis C virus. Alcohol, hepatitis C virus, and MASLD/MASH were approximately equally prevalent as etiologies in non-DM subjects. There was no difference in race/ethnicity or prevalence of varices. Subjects with DM were more likely to use statins (*P* < 0.0001). Although most standard laboratory tests were similar with means in the normal range, means for aspartate transaminase, bilirubin, and Child-Pugh score were lower in DM subjects (Table 4).

**Table 3.** Subject characteristics by type 2 diabetes mellitus diagnosis

	Nondiabetic		Diabetic		*P*-value
	n	Mean ± SD or n (%)	n	Mean ± SD or n (%)	
Age, yr	125	58.8 ± 11.5	130	62.9 ± 9.4	**0.0020**
Male	125	70 (56.0%)	130	57 (43.9%)	0.0695
Weight, kg	125	92.7 ± 25.5	130	98.2 ± 20.5	0.0603
Body mass index, kg m^−2^	125	32.2 ± 7.7	130	34.7 ± 6.4	**0.0043**
Overweight	125	99 (79.2%)	130	124 (95.4%)	**0.0002**
Obese	125	72 (57.6%)	130	93 (71.5%)	**0.0280**
Race					
White	125	111 (88.8%)	130	121 (93.1%)	0.3305
Black or African American	125	10 (8.0%)	130	5 (3.9%)	0.2530
Asian	125	2 (1.6%)	130	0 (0.0%)	0.4606
Other	125	2 (1.6%)	130	4 (3.1%)	0.7154
Ethnicity					
Hispanic	125	16 (12.8%)	130	22 (16.9%)	0.4542
Non-Hispanic	125	109 (87.2%)	130	108 (83.1%)	0.4542
Etiology^a^					
Alcohol-associated liver disease	125	31 (24.8%)	130	7 (5.4%)	**<0.0001**
Autoimmune hepatitis	125	14 (11.2%)	130	3 (2.3%)	**0.0095**
Cryptogenic cirrhosis	125	5 (4.0%)	130	11 (8.5%)	0.2261
Hepatitis B	125	5 (4.0%)	130	2 (1.5%)	0.4126
Hepatitis C	125	44 (35.2%)	130	22 (16.9%)	**0.0014**
Hereditary hemochromatosis	125	1 (0.8%)	130	1 (0.8%)	1.0000
MASLD/MASH	125	35 (28.0%)	130	87 (66.9%)	**<0.0001**
Primary biliary cholangitis	125	3 (2.4%)	130	0 (0.0%)	0.2317
Presence of esophageal varices					
Small varices	116	35 (28.0%)	126	42 (32.3%)	0.6971
Large varices	116	13 (10.4%)	126	15 (11.5%)	1.0000
Antidiabetic and lipid-lowering drugs					
Statins	125	22 (17.6%)	130	74 (56.9%)	**<0.0001**
Metformin	125	4 (3.2%)	130	83 (63.9%)	**<0.0001**
Sulfonylureas	125	0 (0.0%)	130	47 (36.2%)	**<0.0001**
GLP-1 analog	125	0 (0.0%)	130	32 (24.6%)	**<0.0001**
Pioglitazone	125	0 (0.0%)	130	14 (10.8%)	**0.0005**
SGLT-2 inhibitor	125	0 (0.0%)	130	34 (26.2%)	**<0.0001**
DPP-4 inhibitor	125	2 (1.6%)	130	9 (6.9%)	0.0745
Insulin	125	0 (0.0%)	130	49 (37.7%)	**<0.0001**
Vitamin E	125	6 (4.8%)	130	6 (4.6%)	1.0000
Nonselective beta-blockers	125	12 (9.60%)	130	16 (12.31%)	0.6234
Carvedilol	125	4 (3.20%)	130	5 (3.85%)	1.0000
Propranolol	125	6 (4.80%)	130	7 (5.38%)	1.0000
Nadolol	125	2 (1.60%)	130	4 (3.08%)	0.7154

Bold values indicate statistical significance (*P* < 0.05).

MASH, metabolic dysfunction-associated steatohepatitis; MASLD, metabolic dysfunction-associated steatotic liver disease.

a Some subjects have more than 1 etiology of liver disease.

**Table 4.** Laboratory values, clinical scores, and cholate challenge test results by diabetes diagnosis

	Nondiabetic		Diabetic		*P* value
	n	Mean ± SD or n (%)	n	Mean ± SD or n (%)	
Laboratory values					
Albumin, g dL^−1^	125	4.2 ± 0.5	128	4.2 ± 0.4	0.9751
Alk. Phos., U L^−1^	125	104.7 ± 64.7	127	96.8 ± 41.4	0.2458
ALT, U L^−1^	123	41.1 ± 42.2	126	35.2 ± 21.3	0.1603
AST, U L^−1^	119	45.5 ± 29.4	125	38.6 ± 20.1	**0.0322**
Bilirubin, mg dL^−1^	122	0.9 ± 0.5	127	0.7 ± 0.4	**0.0005**
Creatinine, mg dL^−1^	125	0.9 ± 0.3	128	0.9 ± 0.3	0.6013
INR	120	1.1 ± 0.2	125	1.1 ± 0.1	0.1615
Platelets, ×10^3^ μL^-1^	121	154 ± 71	125	155 ± 66	0.8942
Clinical scores					
Child-Pugh score	125	5.2 ± 0.4	130	5.1 ± 0.3	**0.0222**
MELD score	119	8.1 ± 2.3	124	7.6 ± 2.0	0.1071
Cholate challenge test					
DSI	125	23.7 ± 7.7	130	20.9 ± 7.1	**0.0029**
SHUNT%, %	125	41.6 ± 16.0	130	34.4 ± 14.1	**0.0002**
Hepatic reserve, %	125	68.3 ± 19.5	130	75.8 ± 18.1	**0.0015**
HFR_P_, mL min^−1^ kg^−1^	125	10.0 ± 6.4	130	12.6 ± 7.3	**0.0027**
HFR_S_, mL min^−1^ kg^−1^	125	3.3 ± 1.0	130	3.5 ± 0.9	0.1104

Bold values indicate statistical significance (*P* < 0.05).

Alk. Phos., alkaline phosphatase; ALT, alanine transaminase; AST, aspartate transaminase; DSI, disease severity index; HFR_P_, portal hepatic filtration rate; HFR_S_, systemic hepatic filtration rate; INR, international normalized ratio; MELD, model for end-stage liver disease.

Cholate challenge test results in DM vs non-DM subjects are given in Table 4. Parameters of the cholate challenge test indicated better liver function (DSI, hepatic reserve, and HFRs) and less portal-systemic shunting (SHUNT%) in DM subjects.

### Drug treatment

Subjects with either MASLD/MASH or DM diagnoses had better liver function (as judged by lower bilirubin and DSI and higher hepatic reserve) and less severe portal-systemic shunting (as judged by lower SHUNT%). In addition, a large proportion of the MASLD/MASH subjects were also taking maintenance antidiabetic and lipid-lowering drugs, and 57% of DM subjects were taking statins. These findings raised the question of whether the use of these drug therapies was the reason for the better liver function and less shunting observed in MASLD/MASH and DM subjects.

**Figure 1.**
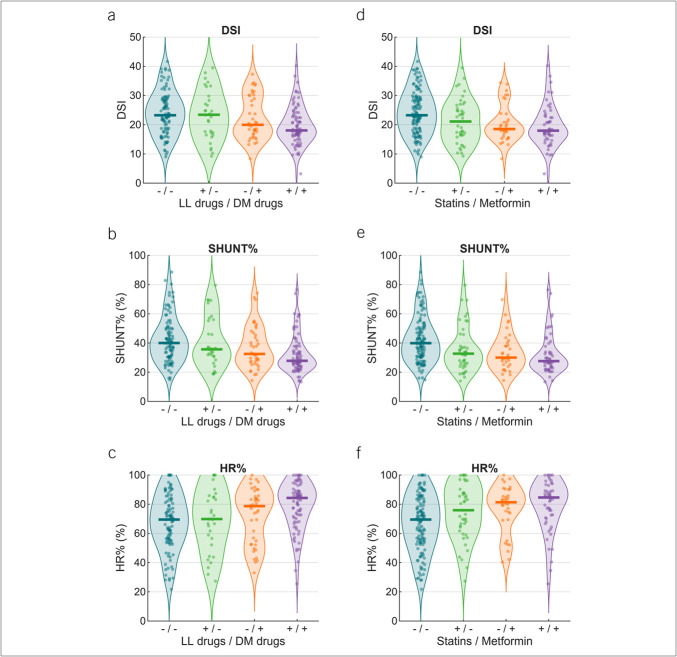
Combined effect of LL drugs and DM drugs on DSI, portal systemic shunting (SHUNT%), and hepatic reserve (HR%) for subjects taking (+) or not taking (−) the drugs. Panels A–C present the results for all LL drugs and DM drugs. Panels D–F present the results for only statins and metformin. DSI, disease severity index; DM, antidiabetic drugs; LL, lipid-lowering.

We compared DSI, SHUNT%, and hepatic reserve across 4 groupings of antidiabetic and lipid-lowering treatments: subjects taking neither (n = 99), subjects taking only antidiabetic drugs (n = 42), subjects taking only lipid-lowering drugs (n = 32), and subjects taking both (n = 82) (Figure 1A–1C). The combination of both antidiabetic and lipid-lowering drugs was associated with the lowest DSI (*P* = 0.002) and SHUNT% (*P* < 0.001) and the highest hepatic reserve (*P* = 0.001). Similar findings were observed with statins and metformin, in subjects taking neither (n = 125), subjects taking only statins (n = 43), subjects taking only metformin (n = 34), and subjects taking both (n = 53) (Figure 1D–1F).

GLP-1 receptor agonists (RAs) are increasingly used not only for treatment of diabetes, but for weight loss, and can slow gastric emptying. Although GLP-1 RAs could theoretically alter the rate of absorption of the oral dose of d4-cholate, we found no difference in clearance curves between subjects taking vs not taking GLP-1 RAs (see Supplementary Figure 2, Supplementary Digital Content, http://links.lww.com/CTG/B453).

### Multivariable analyses

Tables 1 and 3 highlight the most commonly used antidiabetic and lipid-lowering drugs. Vitamin E was included in the list of drugs as a MASH-specific therapy because there was a randomized controlled trial showing its potential benefit and no other drugs were yet approved specifically for MASH treatment at the time of study conduct. Vitamin E was used infrequently, but its use trended higher in MASLD/MASH. In univariable regression, statins, metformin, sulfonylureas, and insulin were the only drugs associated with lower DSI and SHUNT% and greater hepatic reserve. The results of multivariable linear regression including all drugs in prediction of DSI are given in Table 5. Only statins demonstrated statistical significance (*P* = 0.0354), but metformin trended toward significance (*P* = 0.0561). In addition, the coefficients from this analysis indicated that statins alone contributed to a 9.1% lower DSI, and the combination of statins plus metformin to an 18.6% lower DSI. Inclusion of MASLD/MASH and DM diagnoses as predictors did not show evidence of confounding (see Supplementary Table S5, Supplementary Digital Content, http://links.lww.com/CTG/B453). Findings were similar in the associations with both SHUNT% and hepatic reserve (see Supplementary Tables S6 and S7, Supplementary Digital Content, http://links.lww.com/CTG/B453).

**Table 5.** Multivariable regression analysis of the impact of antidiabetic and lipid-lowering drugs and NSBBs on cholate challenge test measurements of DSI

Independent variables	n on drug	Coefficient	SE	*P* value	Resultant DSI	Cumulative reduction in DSI (%)
Base DSI (constant)		24.1			24.1	
Statin	96	−2.2	1.0	**0.0354**	21.9	−9.1%
Metformin	87	−2.3	1.2	**0.0561**	19.6	−18.6%
Sulfonylureas	47	−1.3	1.3	0.3160		
GLP-1 analog	32	2.1	1.6	0.1912		
Pioglitazone	14	0.3	2.3	0.8931		
SGLT-2 inhibitor	34	−0.2	1.6	0.9126		
DPP-4 inhibitor	11	−1.0	2.4	0.6711		
Insulin	49	−1.7	1.3	0.1902		
Vitamin E	12	−0.1	2.2	0.9522		
NSBBs	18^a^	2.0	1.7	0.2550		

All predictors listed were included in the model.

Bold values indicate *P* < 0.10.

DSI, disease severity index; NSBBs, nonselective beta-blockers.

a There were 10 other subjects taking NSBBs for treatment of portal hypertension and varices who were excluded from this analysis.

Taken together, the factors in this analysis associated with better liver function and less portal-systemic shunting were MASLD/MASH diagnosis, DM diagnosis, statin therapy, and metformin therapy. For this reason, we conducted an additional regression analysis of these 4 variables (Table 6). For DSI, metformin was a statistically significant predictor (*P* = 0.046), while statins trended toward significance (*P* = 0.053). Age, MASLD/MASH, and DM were not independently associated with better liver function or less portal-systemic shunting. Similar relationships were found for hepatic reserve and trended for SHUNT%. This suggests that statins and metformin treatments were the major independent variables associated with better liver function and less portal-systemic shunting.

**Table 6.** Multivariable regression analysis of the impact of MASLD/MASH, type 2 DM diagnosis, and drug therapy on cholate challenge test measurements of DSI, SHUNT%, and HR%

Independent variable	Coefficient	SE	*P* value
Impact on DSI (base DSI = 24.9)	Change in DSI		
Statin	−2.1	1.1	**0.0531**
Metformin	−2.6	1.3	**0.0463**
DM diagnosis	−0.4	1.3	0.7649
MASLD/MASH diagnosis	0.0	1.0	0.9740
Age	0.0	0.0	0.7692
Impact on SHUNT% (base SHUNT% = 41.1%)	Change in SHUNT% (%)		
Statin	−3.7	2.2	**0.0892**
Metformin	−4.3	2.6	0.1023
DM diagnosis	−2.4	2.6	0.3684
MASLD/MASH diagnosis	−2.4	2.1	0.2484
Age	0.0	0.1	0.6987
Body mass index	0.0	0.1	0.9562
Impact on HR% (base HR% = 65.4%)	Change in HR% (%)		
Statin	5.1	2.7	**0.0603**
Metformin	6.4	3.2	**0.0507**
DM diagnosis	1.3	3.2	0.6790
MASLD/MASH diagnosis	0.6	2.6	0.8200
Age	0.0	0.1	0.8151

Bold values indicate *P* < 0.10.

DM, diabetes mellitus; DSI, disease severity index; HR%, hepatic reserve; MASH, metabolic dysfunction-associated steatohepatitis; MASLD, metabolic dysfunction-associated steatotic liver disease; SHUNT%, portal-systemic shunting.

## DISCUSSION

Several retrospective studies have suggested potential beneficial effects of statins in CLD, regardless of etiology (5). These benefits might include reduction in portal hypertension, lower rate of decompensating events, lower incidence of hepatocellular carcinoma, lower rate of infectious complications, reduction in fibrosis (action on hepatic stellate cells), and increased survival. However, prospective confirmation of the beneficial effect of statins is lacking. By contrast, the StatLiver trial of 59 patients with cirrhosis and hepatic venous pressure gradient > 10 mm Hg failed to detect either clinical benefit or reduction in portal pressure with 6 months of atorvastatin (13). In addition, the LiverHope trial found no clinical benefit of the combination of simvastatin plus rifaximin in cirrhosis (14). These prospective studies had limitations of relatively small sample sizes, short duration of follow-up, heterogeneity of severity of disease (inclusion of both compensated and decompensated cases), and potential bias in patient selection. For these reasons, 2 large long-term prospective US trials are ongoing to further explore the potential benefit of statins in patients with compensated cirrhosis: LCN RESCU (NCT05832229) and the SACRED Trial (Statins and Cirrhosis: Reducing Events of Decompensation, NCT03654053) (15).

Statins have several effects that may disrupt the pathophysiologic progression of CLD. These include reduction in cholesterol synthesis and lowering of reactive oxidative products of cholesterol, suppression of fibrogenesis by hepatic stellate cells, promotion of endothelial cell vasodilation, and reduction in platelet activation and thrombogenesis. These effects of statins would tend to reduce fibrosis, slow disease progression, and maintain effective sinusoidal perfusion. Our findings of better liver function and less portal-systemic shunting in subjects taking statins are consistent with statin disruption of these pathophysiologic processes and improvement in liver function and shunting. For reference, normal healthy controls without liver disease have DSI <11.6 and SHUNT% <23.7%. Cutoff values for portal hypertension and varices are 18.3 for DSI and 30% for SHUNT%. The DSI and SHUNT% of the statin-treated patients were 20.3 and 33.7%—greater than the normal range, but significantly lower than those of the patients not treated with statins (23.4 and 40.4%, both *P* < 0.01). These differences in DSI and SHUNT% suggest lower risk for clinically significant portal hypertension and varices in the patients taking statins. Patients taking both statins and metformin had even lower DSI and SHUNT% values (19.5 and 32.0%), especially when compared with those not treated with either statins or metformin (24.2 and 42.2%, both *P* < 0.001). Statin therapy spanned both DM vs non-DM cases, and MASH vs non-MASH cases. Thus, our analysis supports a general effect of statins, regardless of CLD etiology.

The oral cholate challenge test is a minimally invasive assessment of the specific liver function of hepatocellular uptake of cholate and the shunting of cholate from portal to systemic circulation. DSI and hepatic reserve score liver function, and SHUNT% quantifies portal-systemic shunting, making the test ideal for uncovering the disruption of hepatocellular function and microcirculation by disease and its modification by treatment. DSI and other test parameters have outperformed standard laboratory tests, other quantitative tests, and clinical models in correlations with stage of fibrosis, likelihood of cirrhosis, likelihood of varices, risk for future clinical outcome, response to drug treatments or other interventions, and in prediction of drug pharmacokinetics in patients with CLD and cirrhosis (6,7,11,16–23). DSI 18.3 is validated as a cutoff in Child-Pugh A cirrhosis for “rule out” of large esophageal varices (6,7), and DSI may have greater sensitivity and lower miss rate in detection of varices than liver stiffness measurement by vibration-controlled transient elastography (see Supplementary Figure 3, Supplementary Digital Content, http://links.lww.com/CTG/B453). The probability of finding large esophageal varices at endoscopy correlates significantly with increasing DSI, SHUNT%, and decreasing hepatic reserve. Furthermore, DSI predicted risk of clinical outcomes in subjects with chronic hepatitis C (18,20), in subjects with compensated or decompensated liver disease (24), and in subjects with primary sclerosing cholangitis (23). These results lend further support to the oral cholate challenge test as a test for effectively quantifying liver function and shunting in advanced liver disease and Child-Pugh A cirrhosis.

In a recent editorial, Kezer, Schmidt, and Shah emphasized the need for additional clinical endpoints for serial monitoring during drug therapy trials in patients with cirrhosis (25). The oral cholate challenge test could fulfill this unmet need.

The SHUNT-V study population was ideal for uncovering drug effects. This cohort of patients was enrolled into the study based on prior selection for the standard of care for screening or surveillance endoscopy (6,7). The population was relatively homogeneous regarding stage of disease, mainly Child-Pugh A cirrhosis. All etiologies of CLD were eligible—about half were MASH and the other half non-MASH. This created an interesting opportunity for comparison of functional differences between etiologies for a given clinical severity of disease, i.e., MASH vs non-MASH. Obesity and diabetes are key drivers of disease severity in MASH (26–29). Because of the significantly greater prevalence of these drivers in MASH, we anticipated that MASH subjects would have more severe disease (higher DSI and higher SHUNT%). In fact, we observed significantly lower DSI and SHUNT% in MASH, and even more surprising, in MASH diabetics. Statins and oral hypoglycemic agents are key treatments in persons with obesity and diabetes. Given the high prevalence of obesity and diabetes in both our MASH and non-MASH groups, we reasoned that the patients receiving these maintenance therapies might be experiencing better long-term lipid and glycemic control which might explain the less hepatic impairment and shunting in MASH and diabetes subjects. Indeed, we found that both statins and antidiabetic drugs were beneficial—functional impairment (DSI) and shunting (SHUNT%) were less in treated subjects, regardless of etiology.

Our analysis also suggests an interaction of statins with antidiabetic drug therapy, particularly metformin. Statins alone were associated with 9.1% lower DSI, but statins plus metformin lowered DSI by 18.6%. The data on metformin as a therapy remain controversial; some studies have suggested benefit, while others have failed. Future studies of statin therapy should examine further the interaction of statins with metformin on clinical trial endpoints.

An interesting observation was that subjects with MASLD/MASH had significantly lower SHUNT% than non-MASH etiologies but nonsignificant differences in other test parameters (Table 2). These results imply that for approximately the same level of liver dysfunction (DSI, HFR, and hepatic reserve), MASLD/MASH may have a lesser degree of portal hypertension and varices risk.

Only a few subjects were taking GLP-1 RAs as a maintenance therapy for diabetes. Although we did not detect an effect of GLP-1 RAs on DSI, SHUNT%, or hepatic reserve, it is clear that GLP-1 RAs may alter gastric emptying. The cholate challenge test depends on the oral dose of d4-cholate to exit the stomach within 60 minutes; delayed gastric emptying may push out the oral absorption curve beyond the 60-minute window. For this reason, in the instructions for use for the cholate challenge tests, GLP-1 RAs should be held for at least 5 days before test performance.

There are limitations to this study. Although the SHUNT-V study was a prospective study comparing results of cholate challenge tests with endoscopic findings, our analysis for potential drug effects is *post hoc*. Despite this drawback, it should be noted that findings from these types of analyses are useful to generate hypotheses, such as those spurring the ongoing LCN-RESCUE and SACRED trials. Taking past retrospective studies into account, our results further support decisions to examine statin therapy as a low-cost, well-tolerated intervention in patients with compensated cirrhosis. Nonetheless, we emphasize that our findings, although intriguing, do not directly link statin treatment with improvement in liver function or reduction in portal systemic shunting. Proof of this effect will require the addition of cholate challenge testing to existing and future prospective trials of statin treatment.

Another limitation is lack of knowledge of reasons for use of statins in the study population, i.e., could bias in selection of patients for statins have contributed to our findings. Perhaps statins were only used in healthier patients over concern of using them in higher risk patients. To address this concern, we have limited our analysis to the 255 patients with Child-Pugh A cirrhosis. In so doing, there were no differences in disease severity between those prescribed vs not prescribed statins as judged by standard laboratory tests, clinical models, and prevalence of varices.

In conclusion, the oral cholate challenge test represents a new tool for uncovering drug effects in patients with CLD. With this tool, we detected a potentially beneficial effect of maintenance use of statins in compensated cirrhosis—preservation of liver function and reduction in portal-systemic shunting, effects that could account for the clinical benefit of statins.

## CONFLICTS OF INTEREST

**Guarantor of the article:** Gregory T. Everson, MD

**Specific author contributions:** G.T.E.: conceived the study concept and design. All co-authors at clinical sites recruited study subjects, performed procedures, and collected data and samples. M.P.Mc., J.K., and G.T.E.: analyzed results, interpreted data, conducted statistical analyses, and drafted and reviewed the manuscript. All cholate analyses were performed in the laboratory of HepQuant, LLC. R.S.R.: investigation; writing—review and editing. E.M.: investigation; writing—review and editing. K.J.L.: investigation; writing—review and editing. A.S.: writing—review and editing. M.P.Mc.: data curation; formal analysis; visualization; writing—original draft; writing—review and editing. J.K.: formal analysis; writing—review and editing. J.C.I.: writing—original draft; writing—review and editing. G.T.E.: conceptualization; methodology; writing—original draft; writing—review and editing.

**Financial support:** The study was sponsored by HepQuant, LLC.

**Potential competing interests:** G. T. Everson, HepQuant, LLC, and the University of Colorado Denver have several issued and pending patents relevant to the HepQuant SHUNT test. G. T. Everson (CEO) and J. C. Imperial (CMO) are equity owners/members of HepQuant, LLC. G. T. Everson and M. P. McRae have pending patents related to the oral cholate challenge test (HepQuant DuO) and simplified dual cholate shunt test (HepQuant SHUNT) versions. M. P. McRae and J. Kittelson are paid consultants for HepQuant, LLC. No other author has a financial relationship to disclose.

**Ethics approval statement:** SHUNT-V was a US multi-center study conducted according to the Declarations of Helsinki and Istanbul. The study was approved by the respective institutional review boards of the participating centers and/or central institutional review board (WIRB).

**Patient consent:** All participants provided written informed consent to participate.

**Clinical trial registration:** The SHUNT-V study was registered at ClinicalTrials.gov (NCT03583996).

**Data availability statement:** Individual patient data will not be shared.

**Study Highlights**

**WHAT IS KNOWN**

✓ Several studies have suggested potential beneficial effects of statins in chronic liver disease.
✓ The oral cholate challenge test is capable of quantifying liver function and physiology.

**WHAT IS NEW HERE**

✓ We evaluated associations of liver disease, diabetes, and maintenance drug therapy in 255 subjects with compensated cirrhosis.
✓ Subjects taking statins and metformin had better liver function and less shunting.
✓ Combined use of these drugs suggests the greatest benefit to liver function.

## Supplementary Material

## ABBREVIATIONS:

CLD: chronic liver disease
DM: diabetes mellitus
DSI: disease severity index
MASH: metabolic dysfunction-associated steatohepatitis
MASLD: metabolic dysfunction-associated steatotic liver disease

## References

[1] American Liver Foundation. About liver disease: how many people have liver disease? https://liverfoundation.org/about-your-liver/facts-about-liver-disease/what-does-your-liver-do/

[2] Tapper EB Parikh ND. Mortality due to cirrhosis and liver cancer in the United States, 1999-2016: Observational study. BMJ 2018;362:k2817. 30021785 10.1136/bmj.k2817 PMC6050518

[3] Hirode G Saab S Wong RJ. Trends in the burden of chronic liver disease among hospitalized US adults. JAMA Netw Open 2020;3(4):e201997–e. 32239220 10.1001/jamanetworkopen.2020.1997 PMC7118516

[4] Mohanty A Tate JP Garcia-Tsao G. Statins are associated with a decreased risk of decompensation and death in veterans with hepatitis C-related compensated cirrhosis. Gastroenterology 2016;150(2):430–40.e1. 26484707 10.1053/j.gastro.2015.10.007 PMC4727998

[5] Marrache MK Rockey DC. Statins for treatment of chronic liver disease. Curr Opin Gastroenterol 2021;37(3):200–7. 33654016 10.1097/MOG.0000000000000716 PMC8691140

[6] Hassanein T Keaveny AP Mantry P . Liver function and portal-systemic shunting quantified by the oral cholate challenge test and risk for large oesophageal varices. Aliment Pharmacol Ther 2024;60(2):246–56. 38778481 10.1111/apt.18054 PMC11348877

[7] Shiffman M Reddy KR Leise MD . Cholate shunt, oral cholate challenge and endoscopic lesions of portal hypertension: The SHUNT-V study. Aliment Pharmacol Ther 2025;61(1):75–87. 39523681 10.1111/apt.18386 PMC11636074

[8] Helmke SM McRae MP Christians U . A validated LC-MS/MS assay for the quantification of cholate isotopes in human serum. J Appl Lab Med 2024;9(6):1028–39. 39150903 10.1093/jalm/jfae094

[9] McRae MP Kittelson J Helmke SM . Advances in noninvasive measurement of liver function and physiology: The HepQuant DuO test. Basic Clin Pharmacol Toxicol 2024;134(3):385–95. 38225212 10.1111/bcpt.13980 PMC13475003

[10] McRae MP Kittelson J Helmke SM, et al. Within-individual reproducibility of a dual sample oral cholate challenge test (DuO) and simplified versions of the HepQuant test. Clin Transl Sci. 2024;17(4):e13786. 38558534 10.1111/cts.13786 PMC10982894

[11] Everson GT Martucci MA Shiffman ML . Portal-systemic shunting in patients with fibrosis or cirrhosis due to chronic hepatitis C: The minimal model for measuring cholate clearances and shunt. Aliment Pharmacol Ther 2007;26(3):401–10. 17635375 10.1111/j.1365-2036.2007.03389.x

[12] McRae MP Helmke SM Burton JR Jr . Compartmental model describing the physiological basis for the HepQuant SHUNT test. Transl Res 2023;252:53–63. 35948199 10.1016/j.trsl.2022.08.002

[13] Kronborg TM Schierwagen R Trošt K . Atorvastatin for patients with cirrhosis. A randomized, placebo-controlled trial. Hepatol Commun 2023;7(12):e0332. 38051553 10.1097/HC9.0000000000000332 PMC10697620

[14] Pose E Napoleone L Amin A . Safety of two different doses of simvastatin plus rifaximin in decompensated cirrhosis (LIVERHOPE-SAFETY): A randomised, double-blind, placebo-controlled, phase 2 trial. Lancet Gastroenterol Hepatol 2020;5(1):31–41. 31607677 10.1016/S2468-1253(19)30320-6

[15] Kaplan DE Mehta R Garcia-Tsao G . SACRED: Effect of simvastatin on hepatic decompensation and death in subjects with high-risk compensated cirrhosis: Statins and cirrhosis: Reducing events of decompensation. Contemp Clin Trials 2021;104:106367. 33771685 10.1016/j.cct.2021.106367 PMC8422958

[16] Everson GT Shiffman ML Morgan TR . The spectrum of hepatic functional impairment in compensated chronic hepatitis C: Results from the hepatitis C anti-viral long-term treatment against cirrhosis trial. Aliment Pharmacol Ther 2008;27(9):798–809. 18266997 10.1111/j.1365-2036.2008.03639.x

[17] Everson GT Shiffman ML Hoefs JC . Quantitative tests of liver function measure hepatic improvement after sustained virological response: Results from the HALT-C trial. Aliment Pharmacol Ther 2009;29(5):589–601. 19053983 10.1111/j.1365-2036.2008.03908.x PMC3767280

[18] Everson GT Shiffman ML Hoefs JC . Quantitative liver function tests improve the prediction of clinical outcomes in chronic hepatitis C: Results from the hepatitis C antiviral long-term treatment against cirrhosis trial. Hepatology 2012;55(4):1019–29. 22030902 10.1002/hep.24752 PMC3298578

[19] Alkhouri N Everson GT Helmke S . Effect of obeticholic acid on liver function in patients with fibrosis due to NASH (abstract). J Hepatol 2019;70(1):e149–e150.

[20] Kittelson J McRae MP Everson GT. Measuring the risk of clinical adverse events (RISK ACE) by quantifying liver function: A patient-centric model. Eur J Intern Med 2025;132:160–3. 39638649 10.1016/j.ejim.2024.11.029 PMC13475004

[21] Lawitz EJ Ertle J Schoelch C . Hepatic improvement within 27 days of avenciguat treatment in Child-Pugh A cirrhosis detected by an oral cholate challenge test. Liver Transpl 2024;30(10):982–90. 38869987 10.1097/LVT.0000000000000420

[22] Kanodia J Giovinazzo H Yates W . Hepatic dysfunction quantified by HepQuant DuO outperforms child-pugh classification in predicting the pharmacokinetics of ampreloxetine. Clin Pharmacol Ther 2024;116(1):186–93. 38654484 10.1002/cpt.3265

[23] Helmke S Kittelson J Imperial JC . The oral cholate challenge test quantifies risk for liver-related clinical outcomes in primary sclerosing cholangitis. Gastro Hep Adv 2024;3(7):944–53. 39286620 10.1016/j.gastha.2024.07.005 PMC11403427

[24] Fallahzadeh MA Hansen DJ Trotter JF . Predicting clinical decompensation in patients with cirrhosis using the HepQuant SHUNT test. Aliment Pharmacol Ther 2021;53(8):928–38. 33556192 10.1111/apt.16283

[25] Kezer CA Schmidt KA Shah VH. Statin the course: Navigating unchartered territory in cirrhosis. Hepatol Commun 2024;8(6):e0456. 38829204 10.1097/HC9.0000000000000456 PMC11150024

[26] Cernea S. NAFLD fibrosis progression and type 2 diabetes: The hepatic–metabolic interplay. Life 2024;14(2):272. 38398781 10.3390/life14020272 PMC10890557

[27] Rhee E-J. Nonalcoholic fatty liver disease and diabetes: An epidemiological perspective. Endocrinol Metab 2019;34(3):226–33. 10.3803/EnM.2019.34.3.226 PMC6769345 31565874

[28] Tilg H Moschen AR Roden M. NAFLD and diabetes mellitus. Nat Rev Gastroenterol Hepatol 2017;14(1):32–42. 27729660 10.1038/nrgastro.2016.147

[29] Fujii H Kawada N. Japan Study Group Of NAFLD (JSG-NAFLD). The role of insulin resistance and diabetes in nonalcoholic fatty liver disease. Int J Mol Sci. 2020;21(11):3863. 32485838 10.3390/ijms21113863 PMC7312931

