# Supplementary material for: HMG-CoA Reductase Inhibitors (Statins) May Preserve Hepatic Function and Reduce Portal-Systemic Shunting in Compensated Advanced Chronic Liver Disease: Results From the SHUNT-V Study
Source: Clin Transl Gastroenterol. 2026 Jan 26;17(3):e00980. doi: 10.14309/ctg.0000000000000980 (PMC13008230; doi:10.14309/ctg.0000000000000980)
Supplement: Supplementary file 1 [file ct9-17-e00980-s001.docx]

# Supplemental Materials


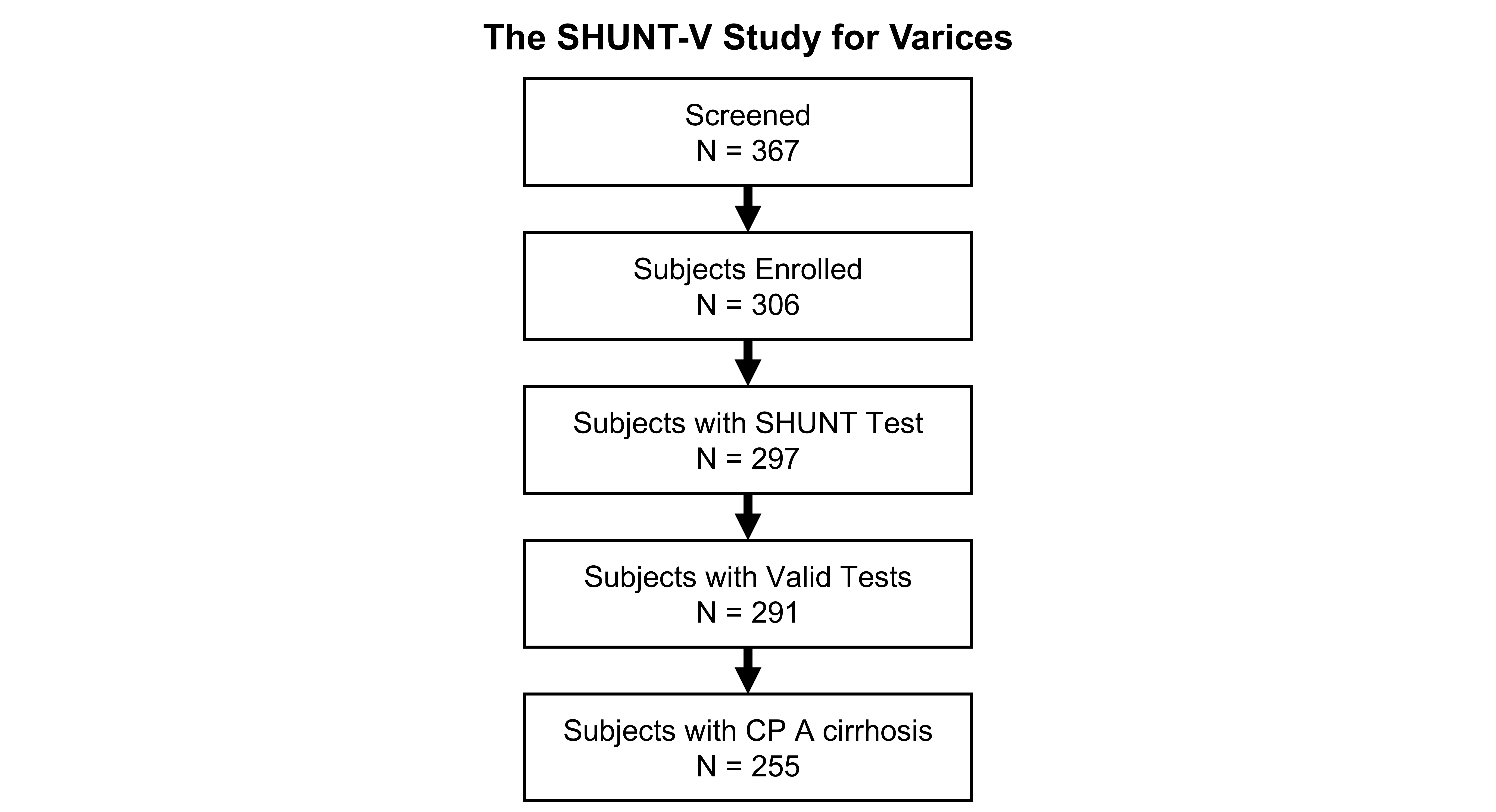


**Figure S1.** Disposition of study subjects

| **A**  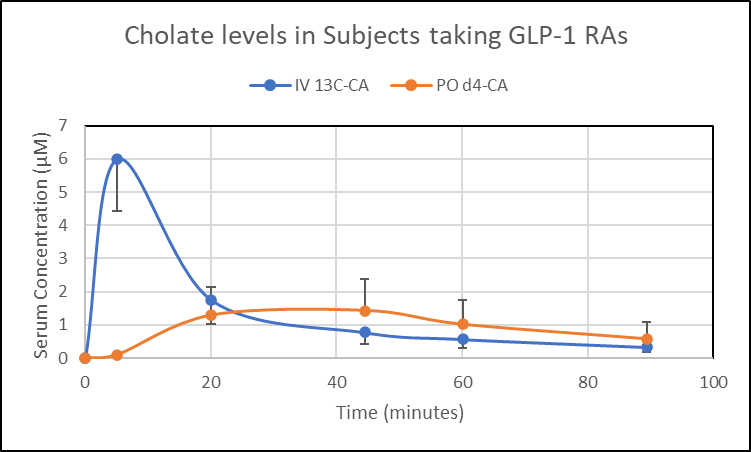 |
| --- |
| **B**  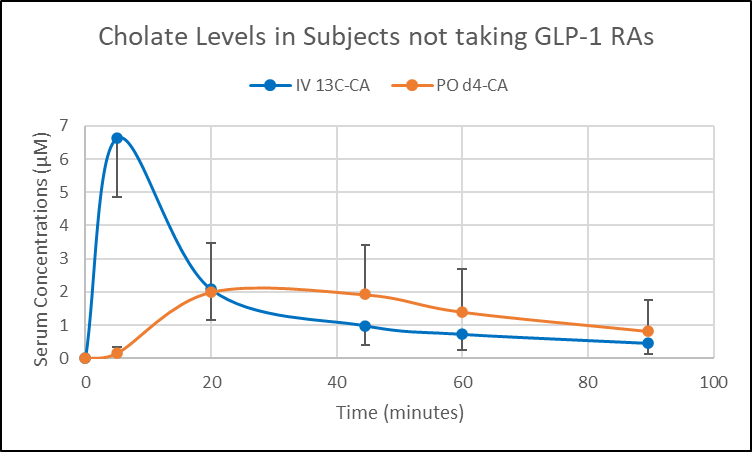 |

**Figure S2.** Time-dependent pattern of absorption, peak concentration, and elimination, as reflected in the serum concentrations of d4-cholate, was similar between groups of subjects taking (Panel A, n=32) and not taking (Panel B, n=243) maintenance GLP-1s for Type 2 diabetes.

| **A.** | **DSI Captured More Endoscopic Lesions of Portal Hypertension than LSM**  **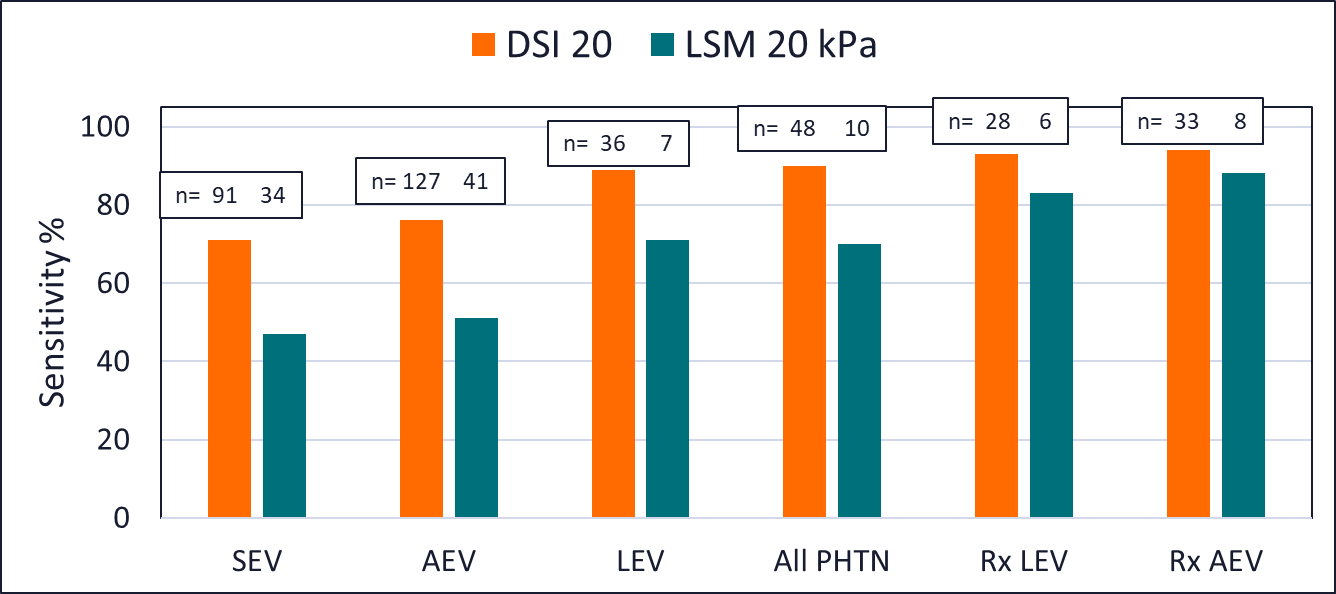** |
| --- | --- |
| **B.** | **DSI Missed Fewer Varices than LSM**  **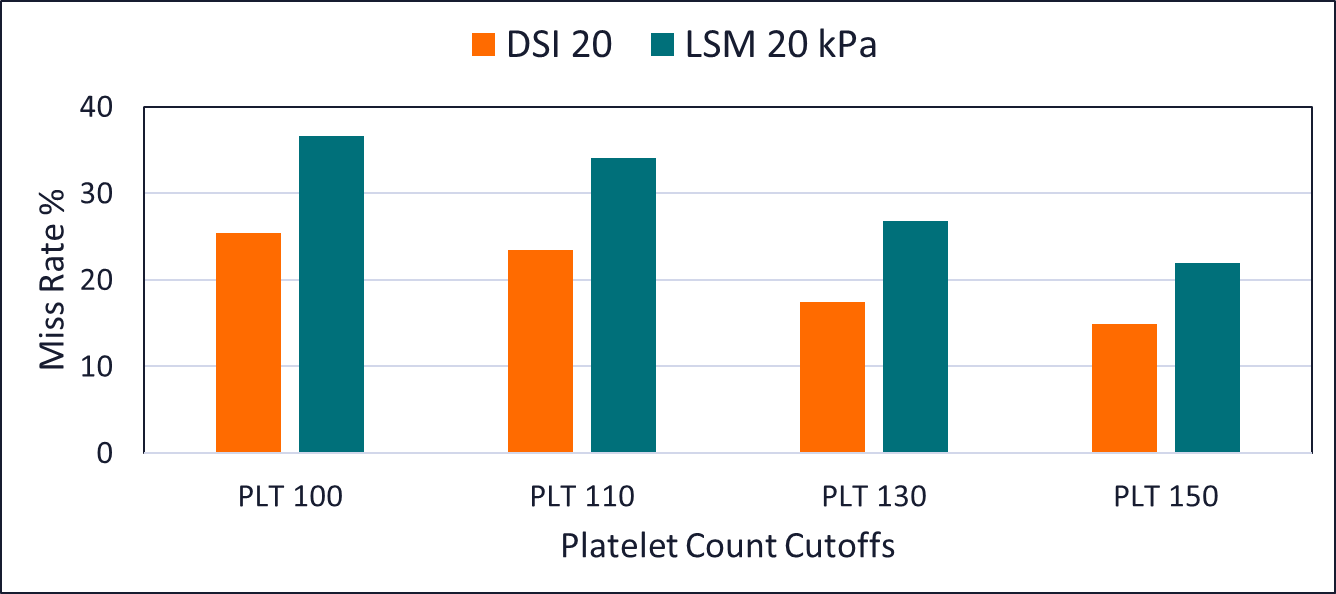** |

**Figure S3.** Comparison of Disease Severity Index (DSI, n=275) from the oral cholate challenge test to liver stiffness measurements (LSM, n=86) for diagnostic sensitivity in detecting endoscopic lesions of portal hypertension (A) and miss rate for any esophageal varices at various platelet count cutoffs used in the Baveno criteria (B). For Panel B, results were similar for DSI vs LSM comparisons of 18 vs 19, 20 vs 20, and 23 vs 25 where EGD avoidance rates were equivalently 38%, 43%, and 55%, respectively. Abbreviations: DSI: disease severity index from HepQuant DuO test; LSM: liver stiffness measurement by vibration controlled transient elastography; SEV: small esophageal varices; AEV: any esophageal varices; LEVs: large esophageal varices; Rx LEV: banded at EGD or varices medication prescribed post-EGD; Rx AEV: Treated Any EVs included large and small varices that were treated; All PHTN: all significant endoscopic lesions of portal hypertension (LEV, Rx LEV, Rx AEV, large gastric varices, varices with red wale signs and severe portal hypertensive gastropathy).

**Table S1.** Subject characteristics – all subjects

|  | **All Subjects** | |
| --- | --- | --- |
|  | n | Mean ± SD or n (%) |
| Age, years | 255 | 61 ± 11 |
| Male | 255 | 127 (49.8%) |
| Weight, kg | 255 | 95.5 ± 23.2 |
| Body mass index, kg m^−2^ | 255 | 33.5 ± 7.2 |
| Overweight | 255 | 223 (87.5%) |
| Obese | 255 | 165 (64.7%) |
| Diabetes, n (%) | 255 | 130 (51.0%) |
| Race |  |  |
| White | 255 | 232 (91.0%) |
| Black or African American | 255 | 15 (5.9%) |
| Asian | 255 | 2 (0.8%) |
| Other | 255 | 6 (2.4%) |
| Ethnicity |  |  |
| Hispanic | 255 | 38 (14.9%) |
| Non-Hispanic | 255 | 217 (85.1%) |
| Etiology^†^ |  |  |
| Alcohol-associated liver disease | 255 | 38 (14.9%) |
| Autoimmune hepatitis | 255 | 17 (6.7%) |
| Cryptogenic cirrhosis | 255 | 16 (6.3%) |
| Hepatitis B | 255 | 7 (2.8%) |
| Hepatitis C | 255 | 66 (25.9%) |
| Hereditary haemochromatosis | 255 | 2 (0.8%) |
| MASLD/MASH | 255 | 122 (47.8%) |
| Primary biliary cholangitis | 255 | 3 (1.2%) |
| Presence of esophageal varices |  |  |
| Small varices | 242 | 77 (31.8%) |
| Large varices | 242 | 28 (11.6%) |
| Antidiabetic & lipid-lowering drugs |  |  |
| Statins | 255 | 96 (37.7%) |
| Metformin | 255 | 87 (34.1%) |
| Sulfonylureas | 255 | 47 (18.4%) |
| GLP-1 Analogue | 255 | 32 (12.6%) |
| Pioglitazone | 255 | 14 (5.5%) |
| SGLT-2 Inhibitor | 255 | 34 (13.3%) |
| DPP-4 Inhibitor | 255 | 11 (4.3%) |
| Insulin | 255 | 49 (19.2%) |
| Vitamin E | 255 | 12 (4.7%) |

^†^ Some subjects have more than one etiology of liver disease

**Table S2.** Laboratory tests, clinical scores, and oral cholate challenge test (HepQuant DuO) results for all subjects.

|  | **All Subjects** | |
| --- | --- | --- |
|  | n | Mean ± SD or n (%) |
| Laboratory Values |  |  |
| Albumin, g dL^−1^ | 253 | 4.24 ± 0.42 |
| Alk. Phos., U L^−1^ | 252 | 100.69 ± 54.26 |
| ALT, U L^−1^ | 249 | 38.12 ± 33.34 |
| AST, U L^−1^ | 244 | 41.98 ± 25.25 |
| Bilirubin, mg dL^−1^ | 249 | 0.76 ± 0.46 |
| Creatinine, mg dL^−1^ | 253 | 0.90 ± 0.29 |
| INR | 245 | 1.09 ± 0.14 |
| Platelets, ×10^3^ µL^-1^ | 246 | 154.09 ± 68.20 |
| Clinical Scores |  |  |
| Child-Pugh score | 255 | 5.14 ± 0.35 |
| MELD score | 243 | 7.84 ± 2.20 |
| Oral cholate challenge test |  |  |
| DSI | 255 | 22.25 ± 7.51 |
| SHUNT%, % | 255 | 37.89 ± 15.46 |
| Hepatic Reserve, % | 255 | 72.13 ± 19.13 |
| HFR_P_, mL min^−1^ kg^−1^ | 255 | 11.33 ± 6.99 |
| HFR_S_, mL min^−1^ kg^−1^ | 255 | 3.41 ± 0.94 |

Abbreviations: Alk. Phos. = alkaline phosphatase; ALT = alanine transaminase; AST = aspartate transaminase; DSI = Disease Severity Index; HFR_P_ = Portal Hepatic Filtration Rate; HFR_S_ = Systemic Hepatic Filtration Rate; INR = International normalized ratio; MELD = Model for End-Stage Liver Disease.

**Table S3.** Results with various HepQuant SHUNT test versions by MASLD/MASH

| **Test Version / Test Parameter** | **All Subjects** | | **Other chronic liver disease etiologies** | | **MASLD/MASH** | | **p-value** |
| --- | --- | --- | --- | --- | --- | --- | --- |
|  | **n** | **Mean ± SD or n (%)** | **n** | **Mean ± SD or n (%)** | **n** | **Mean ± SD or n (%)** |  |
| SHUNT V1.0 |  |  |  |  |  |  |  |
| DSI | 254 | 23.14 ± 7.36 | 132 | 23.75 ± 7.92 | 122 | 22.49 ± 6.67 | 0.1715 |
| SHUNT%, % | 254 | 38.69 ± 17.03 | 132 | 40.27 ± 17.35 | 122 | 37.00 ± 16.57 | 0.1265 |
| Hepatic Reserve, % | 254 | 70.45 ± 18.99 | 132 | 68.82 ± 20.39 | 122 | 72.21 ± 17.27 | 0.1560 |
| HFR_P_, mL min^−1^ kg^−1^ | 255 | 10.95 ± 6.69 | 133 | 10.57 ± 7.08 | 122 | 11.37 ± 6.24 | 0.3370 |
| HFR_S_, mL min^−1^ kg^−1^ | 254 | 3.36 ± 1.02 | 132 | 3.33 ± 1.08 | 122 | 3.39 ± 0.94 | 0.6507 |
| SHUNT V1.1 |  |  |  |  |  |  |  |
| DSI | 255 | 22.71 ± 7.48 | 133 | 23.33 ± 8.05 | 122 | 22.04 ± 6.79 | 0.1687 |
| SHUNT%, % | 255 | 39.54 ± 16.90 | 133 | 41.43 ± 17.66 | 122 | 37.49 ± 15.86 | 0.0631 |
| Hepatic Reserve, % | 255 | 70.94 ± 19.29 | 133 | 69.18 ± 20.56 | 122 | 72.87 ± 17.70 | 0.1272 |
| HFR_P_, mL min^−1^ kg^−1^ | 255 | 10.92 ± 6.73 | 133 | 10.53 ± 7.12 | 122 | 11.34 ± 6.28 | 0.3401 |
| HFR_S_, mL min^−1^ kg^−1^ | 255 | 3.41 ± 0.94 | 133 | 3.39 ± 1.04 | 122 | 3.42 ± 0.82 | 0.8436 |
| SHUNT V2.0 |  |  |  |  |  |  |  |
| DSI | 255 | 22.43 ± 7.38 | 133 | 23.19 ± 7.91 | 122 | 21.61 ± 6.71 | 0.0877 |
| SHUNT%, % | 255 | 38.12 ± 16.61 | 133 | 40.36 ± 17.22 | 122 | 35.69 ± 15.62 | **0.0245** |
| Hepatic Reserve, % | 255 | 71.78 ± 18.92 | 133 | 69.63 ± 20.06 | 122 | 74.12 ± 17.38 | 0.0581 |
| HFR_P_, mL min^−1^ kg^−1^ | 255 | 11.33 ± 6.99 | 133 | 10.76 ± 7.34 | 122 | 11.94 ± 6.56 | 0.1778 |
| HFR_S_, mL min^−1^ kg^−1^ | 255 | 3.40 ± 0.95 | 133 | 3.38 ± 1.05 | 122 | 3.42 ± 0.84 | 0.7490 |

Bold values indicate statistical significance (p < 0.05)

Abbreviations: DSI = Disease Severity Index; HFR_P_ = Portal Hepatic Filtration Rate; HFR_S_ = Systemic Hepatic Filtration Rate.

**Table S4.** Results with various HepQuant SHUNT test versions by diabetes mellitus diagnosis

| **Test Version / Test Parameter** | **All Subjects** | | **Non-diabetic** | | **Diabetic** | | **p-value** |
| --- | --- | --- | --- | --- | --- | --- | --- |
|  | **n** | **Mean ± SD or n (%)** | **n** | **Mean ± SD or n (%)** | **n** | **Mean ± SD or n (%)** |  |
| SHUNT V1.0 |  |  |  |  |  |  |  |
| DSI | 254 | 23.14 ± 7.36 | 124 | 24.51 ± 7.60 | 130 | 21.84 ± 6.90 | **0.0037** |
| SHUNT%, % | 254 | 38.69 ± 17.03 | 124 | 41.14 ± 17.02 | 130 | 36.36 ± 16.77 | **0.0248** |
| Hepatic Reserve, % | 254 | 70.45 ± 18.99 | 124 | 66.91 ± 19.69 | 130 | 73.83 ± 17.73 | **0.0035** |
| HFR_P_, mL min^−1^ kg^−1^ | 255 | 10.95 ± 6.69 | 125 | 9.78 ± 6.15 | 130 | 12.08 ± 7.01 | **0.0059** |
| HFR_S_, mL min^−1^ kg^−1^ | 254 | 3.36 ± 1.02 | 124 | 3.21 ± 1.05 | 130 | 3.50 ± 0.97 | **0.0241** |
| SHUNT V1.1 |  |  |  |  |  |  |  |
| DSI | 255 | 22.71 ± 7.48 | 125 | 24.04 ± 7.76 | 130 | 21.43 ± 7.00 | **0.0052** |
| SHUNT%, % | 255 | 39.54 ± 16.90 | 125 | 42.50 ± 16.89 | 130 | 36.70 ± 16.48 | **0.0059** |
| Hepatic Reserve, % | 255 | 70.94 ± 19.29 | 125 | 67.42 ± 19.99 | 130 | 74.32 ± 18.04 | **0.0041** |
| HFR_P_, mL min^−1^ kg^−1^ | 255 | 10.92 ± 6.73 | 125 | 9.74 ± 6.17 | 130 | 12.04 ± 7.07 | **0.0061** |
| HFR_S_, mL min^−1^ kg^−1^ | 255 | 3.41 ± 0.94 | 125 | 3.30 ± 1.00 | 130 | 3.51 ± 0.87 | 0.0752 |
| SHUNT V2.0 |  |  |  |  |  |  |  |
| DSI | 255 | 22.43 ± 7.38 | 125 | 23.84 ± 7.63 | 130 | 21.07 ± 6.90 | **0.0026** |
| SHUNT%, % | 255 | 38.12 ± 16.61 | 125 | 41.29 ± 16.41 | 130 | 35.08 ± 16.28 | **0.0027** |
| Hepatic Reserve, % | 255 | 71.78 ± 18.92 | 125 | 67.99 ± 19.44 | 130 | 75.42 ± 17.74 | **0.0016** |
| HFR_P_, mL min^−1^ kg^−1^ | 255 | 11.33 ± 6.99 | 125 | 9.99 ± 6.41 | 130 | 12.61 ± 7.30 | **0.0027** |
| HFR_S_, mL min^−1^ kg^−1^ | 255 | 3.40 ± 0.95 | 125 | 3.29 ± 1.00 | 130 | 3.50 ± 0.89 | 0.0731 |

Bold values indicate statistical significance (p < 0.05)

Abbreviations: DSI = Disease Severity Index; HFR_P_ = Portal Hepatic Filtration Rate; HFR_S_ = Systemic Hepatic Filtration Rate.

**Table S5.** Multiple regression analysis of the impact of antidiabetic and lipid lowering drugs, and MASLD/MASH and DM diagnoses, on oral cholate challenge test (HepQuant DuO) measurements of disease severity index (DSI)

| **Independent variables** | **n on drug** | **Coefficient** | **SE** | **p Value** | **Resultant DSI** | **Cumulative Reduction in DSI (%)** |
| --- | --- | --- | --- | --- | --- | --- |
| Base DSI (constant) |  | 23.9 |  |  | 23.9 |  |
| Statin | 96 | -2.3 | 1.1 | **0.0340** | 21.6 | -9.6% |
| Metformin | 87 | -2.4 | 1.3 | **0.0722** | 19.2 | -19.7% |
| Sulfonylureas | 47 | -1.4 | 1.4 | 0.2993 |  |  |
| GLP-1 Analogue | 32 | 2.0 | 1.6 | 0.2063 |  |  |
| Pioglitazone | 14 | 0.3 | 2.3 | 0.8922 |  |  |
| SGLT-2 Inhibitor | 34 | -0.2 | 1.6 | 0.8855 |  |  |
| DPP-4 Inhibitor | 11 | -1.1 | 2.4 | 0.6372 |  |  |
| Insulin | 49 | -1.8 | 1.4 | 0.1872 |  |  |
| Vitamin E | 12 | -0.2 | 2.2 | 0.9197 |  |  |
| NSBBs | 18 ^a^ | 2.0 | 1.7 | 0.2477 |  |  |
| MASLD/MASH diagnosis | - | 0.4 | 1.0 | 0.7022 |  |  |
| DM diagnosis | - | 0.2 | 1.4 | 0.9062 |  |  |

Bold values indicate p<0.10

^a^ There were 10 other subjects taking NSBBs for treatment of portal hypertension and varices who were excluded from this analysis

**Table S6.** Multiple regression analysis of the impact of antidiabetic and lipid lowering drugs on oral cholate challenge test (HepQuant DuO) estimates of portal-systemic shunting (SHUNT%)

| **Independent variables** | **n on drug** | **Coefficient** | **SE** | **p Value** | **Resultant SHUNT%** | **Cumulative Reduction in SHUNT% (%)** |
| --- | --- | --- | --- | --- | --- | --- |
| Base SHUNT% (constant) |  | 42.0 |  |  | 42.0% |  |
| Statin | 96 | -4.6 | 2.1 | **0.0301** | 37.3% | 11.0% |
| Metformin | 87 | -4.3 | 2.4 | **0.0816** | 33.1% | 21.1% |
| Sulfonylureas | 47 | -2.6 | 2.7 | 0.3406 |  |  |
| GLP-1 Analogue | 32 | 0.7 | 3.2 | 0.8264 |  |  |
| Pioglitazone | 14 | -1.0 | 4.7 | 0.8355 |  |  |
| SGLT-2 Inhibitor | 34 | -1.6 | 3.2 | 0.6198 |  |  |
| DPP-4 Inhibitor | 11 | -4.3 | 4.9 | 0.3821 |  |  |
| Insulin | 49 | -2.8 | 2.6 | 0.2811 |  |  |
| Vitamin E | 12 | -1.6 | 4.5 | 0.7197 |  |  |
| NSBBs | 18 ^a^ | 7.6 | 3.5 | **0.0320** |  |  |

Bold values indicate p<0.10

^a^ There were 10 other subjects taking NSBBs for treatment of portal hypertension and varices who were excluded from this analysis

**Table S7.** Multiple regression analysis of the impact of antidiabetic and lipid lowering drugs on oral cholate challenge test (HepQuant DuO) measurements of hepatic reserve (HR%)

| **Independent variables** | **n on drug** | **Coefficient** | **SE** | **p Value** | **Resultant HR%** | **Cumulative Improvement in HR% (%)** |
| --- | --- | --- | --- | --- | --- | --- |
| Base HR% (constant) |  | 67.4 |  |  | 67.4 |  |
| Statin | 96 | 5.6 | 2.6 | **0.0359** | 73.0 | 8.3% |
| Metformin | 87 | 5.9 | 3.0 | **0.0538** | 78.8 | 17.0% |
| Sulfonylureas | 47 | 3.7 | 3.4 | 0.2794 |  |  |
| GLP-1 Analogue | 32 | -4.6 | 4.0 | 0.2516 |  |  |
| Pioglitazone | 14 | -0.3 | 5.9 | 0.9658 |  |  |
| SGLT-2 Inhibitor | 34 | 1.0 | 4.0 | 0.8099 |  |  |
| DPP-4 Inhibitor | 11 | 2.2 | 6.1 | 0.7213 |  |  |
| Insulin | 49 | 3.8 | 3.3 | 0.2425 |  |  |
| Vitamin E | 12 | 0.5 | 5.6 | 0.9244 |  |  |
| NSBBs | 18 ^a^ | -5.5 | 4.4 | 0.2132 |  |  |

Bold values indicate p<0.10

^a^ There were 10 other subjects taking NSBBs for treatment of portal hypertension and varices who were excluded from this analysis
